# Melatonin Receptor Genes in Vertebrates

**DOI:** 10.3390/ijms140611208

**Published:** 2013-05-27

**Authors:** Di Yan Li, David Glenn Smith, Rüdiger Hardeland, Ming Yao Yang, Huai Liang Xu, Long Zhang, Hua Dong Yin, Qing Zhu

**Affiliations:** 1 College of Animal Science and Technology, Sichuan Agricultural University, Ya’an 625014, China; E-Mails: lidiyan860714@163.com (D.Y.L.); yanghomeuk2000@yahoo.co.uk (M.Y.Y.); xuhuail@yahoo.com.cn (H.L.X.); zlong4723@yahoo.com.cn (L.Z.); yin881986@yahoo.com.cn (H.D.Y.); 2 Department of Anthropology and California National Primate Research Center, University of California, Davis, CA 95616, USA; E-Mail: dgsmith@ucdavis.edu; 3 Institute of Zoology and Anthropology, University of Goettingen, Berliner Str. 28, Goettingen D-37073, Germany; E-Mail: rhardel@gwdg.de

**Keywords:** melatonin receptor, evolution, vertebrates

## Abstract

Melatonin receptors are members of the G protein-coupled receptor (GPCR) family. Three genes for melatonin receptors have been cloned. The *MT1* (or *Mel1a* or *MTNR1A*) and MT2 (or *Mel1b* or *MTNR1B*) receptor subtypes are present in humans and other mammals, while an additional melatonin receptor subtype, *Mel1c* (or *MTNR1C*), has been identified in fish, amphibians and birds. Another melatonin related orphan receptor, *GPR50*, which does not bind melatonin, is found exclusively in mammals. The hormone melatonin is secreted primarily by the pineal gland, with highest levels occurring during the dark period of a circadian cycle. This hormone acts systemically in numerous organs. In the brain, it is involved in the regulation of various neural and endocrine processes, and it readjusts the circadian pacemaker, the suprachiasmatic nucleus. This article reviews recent studies of gene organization, expression, evolution and mutations of melatonin receptor genes of vertebrates. Gene polymorphisms reveal that numerous mutations are associated with diseases and disorders. The phylogenetic analysis of receptor genes indicates that *GPR50* is an outgroup to all other melatonin receptor sequences. *GPR50* may have separated from a melatonin receptor ancestor before the split between *MTNR1C* and the *MTNR1A*/B ancestor.

## 1. Introduction

In vertebrates, melatonin (*N*-acetyl-5-methoxytryptamine), regulates various biological functions through three different subtypes of G protein-coupled receptors (GPCRs), Mel1a (alias MT1, *MTNR1A*), Mel1b (alias MT2, *MTNR1B*), and Mel1c (*MTNR1C*) [1–3]. The contribution of several other binding proteins to melatonin signaling is still controversial [4] and will not be considered in this article. The *MTNR1A* and *MTNR1B* receptor subtypes, encoded by genes on human chromosomes 4 and 11, respectively, are present in humans and other mammals, while an additional melatonin receptor subtype, *MTNR1C*, has been identified in fish, amphibians and birds. A related protein, *GPR50*, expressed in eutherian mammals, has been originally interpreted as an ortholog of the nonmammalian *MTNR1C* [5] and is usually regarded as an orphan GPCR, which does not bind melatonin [6], and for which no other low-molecular weight ligand is known to date. It shares 45% identity with the melatonin receptor family [6]. *GPR50* is encoded by a gene located on the X chromosome (Xq28) [7] and especially expressed in the pars intermedia of the pituitary, in hypothalamus and hippocampus [8]. *GPR50* has been shown to heterodimerize with both *MTNR1A* and *MTNR1B* receptors, but interferes only with *MTNR1A* signaling [9]. Deletion of the large *C*-terminal tail of *GPR50* abolishes the inhibitory effect of *GPR50* on *MTNR1A* without affecting heterodimerization, indicating that this domain interacts with *MTNR1A*, but not *MTNR1B*, or mediates interactions with other regulatory proteins [9]. *GPR50* has not been found in fish or birds [5]. Evolutionary studies have provided evidence that the *GPR50* group evolved under different selective pressure than the orthologous groups *MTNR1A*, B, and C [5]. Melatonin, acting through melatonin receptors, is involved in numerous physiological processes including blood pressure regulation [10], circadian entrainment [11], retinal physiology [12,13], oncogenesis [12], seasonal reproduction [14], ovarian physiology [15], and osteoblast differentiation [16] (for further details and receptor distribution see [4]).

Many factors contribute to the diversity of the melatonin response within the body [17]. First, melatonin levels fluctuate within the circadian cycle [17,18] and throughout the year [18,19]. Levels of melatonin are lowest during the day and highest at night and the nocturnal maxima are broader during winter than summer. Temporal patterns of receptor expression and affinity do not necessarily follow the rhythm of the circulating hormone. In this context, receptor downregulation and internalization have to be also considered. The rhythm of the *MTNR1C* receptor in chicks is opposite to that of *MTNR1A* and *MTNR1B*, with higher levels occurring during the day than at night [20,21]. Second, melatonin can activate or inhibit other signal transduction cascades. Additionally, receptor-independent actions of melatonin are known, especially in the context of antioxidant [22] and, perhaps, hypnotic actions [23]. This would require uptake into cells, which seems to be facilitated by the amphiphilic nature of this small molecule, which crosses membranes with ease [24,25], or by active uptake mechanisms [26]. The melatonin-activated GPCRs can couple to multiple signal transduction cascades, either alternately, or concomitantly in the same tissue [27]. Third, melatonin receptor expression, and perhaps function, can be regulated by multiple cues including the light/dark cycle [28], scheduled arousal, an endogenous pacemaker, melatonin itself, and/or other hormones [17,29].

## 2. Expression of Melatonin Receptors

Melatonin receptors are found in several central nervous and numerous peripheral tissues [4]. A specific aspect that has received particular attention concerns the melatonergic modulation of hypothalamic-pituitary-gonadal axis, which is of major importance in seasonal breeders, but also exists in variant forms in animals without seasonally restricted reproduction, including the human [30]. Expression of melatonin-related receptor mRNA in rodents has been identified, at highest levels in the suprachiasmatic nuclei (SCN), but also in other brain regions, including parts of the preoptic area, parabrachial nuclei, olfactory bulb, prefrontal cortex, cerebellar cortex, hippocampus, basal ganglia, substantia nigra, ventral tegmental area, nucleus accumbens and retina, in brain-associated tissues, at highest density in the pars tuberalis, and in the choroid plexus, as well as peripheral organs such as kidney, adrenal gland, intestine, stomach, heart, lung, skin, testis and ovary [4,31]. This pattern of distribution strongly suggests a conserved function in neuroendocrine regulation and a role in the orchestration of physiological responses and rhythms in both the central nervous system and peripheral tissues [4,31]. Melatonin receptors in humans have been detected in the SCN, in various other parts of the hypothalamus and additional brain areas, such as paraventricular nucleus, periventricular nucleus, supraoptic nucleus, sexually dimorphic nucleus, the diagonal band of Broca, the nucleus basalis of Meynert, infundibular nucleus, ventromedial and dorsomedial nuclei, tuberomammillary nucleus, mammillary bodies, hippocampus, amygdala, substantia nigra, paraventricular thalamic nucleus, cortical areas, cerebellar cortex–including expression in Bergmann glia and other astrocytes, in retina, cardiovascular system, the gastrointestinal tract, parotid gland, exocrine and endocrine pancreas, liver and gallbladder, kidney, immune cells, adipocytes, prostate and breast epithelial cells, ovary/granulosa cells, myometrium, and skin [4,32]. However, there is considerable variation in the density and location of the expression of melatonin receptors between species [33]. Correspondingly, *MTNR1C* is also expressed in various areas of the brain of many nonmammalian vertebrates [34]. Melatonin regulates circadian rhythms, hibernation, feeding pattern, thermoregulation, and neuroendocrine functions of birds [35]. Especially in seasonal breeders, melatonin is involved in ovarian function by activating multiple receptors and signaling pathways on different target cell types, especially theca and granulosa cells [36]. While melatonin receptors are found almost everywhere in the human body, many aspects of melatonin’s functional role in humans remain to be elucidated, except for its circadian, temperature-regulating, sleep promoting and some vascular effects [32]. Some caution is due because expression studies were often only based on the mRNA and not also the protein level.

*GPR50* has been detected in hypothalamo-pituitary regions of mammals, including the pars tuberalis of humans [6] and sheep [37], the dorsomedial hypothalamus of rodents [38], and, at high expression levels, in the ependymal cell layer of the third ventricle of all species examined [39]. Its deviatant pattern of expression, its interaction with *MTNR1A* and the lack of affinity for melatonin are in favor of a regional-specific modulation of melatonin signaling [5]. However, it should be noted that *GPR50* has obviously additional functions not related to melatonin. It was found to also interact with the neurite outgrow inhibitor NOGO-A [40] and with TIP60, a coactivator of glucocorticoid receptor signaling and histone acetyltransferase [41]. Several of the metabolic changes observed in *GPR50* knockouts [38] may, thus, be attributable to disturbances of functions different from melatonin signaling.

## 3. Polymorphisms of Human Melatonin Receptor and *GPR50* Genes

**Figure 1:**
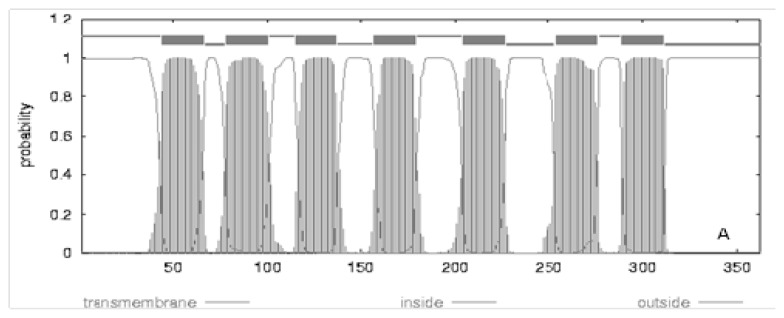

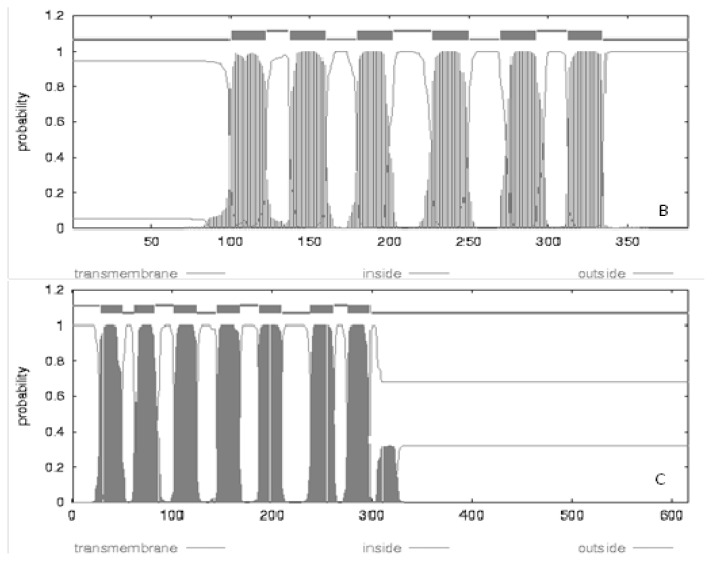
Seven-transmembrane structure of typical melatonin receptors from most vertebrate species (panel **A**), and deviations of cat *MTNR1A* (panel **B**) and *GPR50* (panel **C**). Sequences were examined by a transmembrane protein topology prediction method based on a hidden Markov model (TMHMM) [55] for the presence of seven trans-membrane domains.

Melatonin regulates circadian rhythms through feedback to the SCN, the central biological clock of the brain [42–44]. In addition to these relatively well understood mechanisms, evidence has accumulated for concomitant actions on non-SCN oscillators in the central nervous system and in peripheral organs [45]. *MTNR1A* and *MTNR1B* encode high affinity receptors whose sequences encode 351 and 363 amino acids, respectively, whereas *GPR50* is composed of 618 amino acids with 7TM hydrophobic segments (Figure 1, panel A). The principal features of *GPR50* include a long *C*-tail (Figure 1, panel C) of over 300 amino acids and the absence of consensus sites for *N*-linked glycosylation in either the amino terminus or the predicted extracellular loops [8]. Human polymorphisms of all three genes are summarized in Table 1. Dysfunction of endogenous clocks, melatonin receptor polymorphisms, and age-associated decline of melatonin probably contribute to numerous diseases including cancer, metabolic syndrome, diabetes type 2, hypertension, and several mood and cognitive disorders [45]. Expression of melatonin receptors and its variants in tumor cell lines and in animal models has been reported to be relevant to breast cancer [46,47], invasive ductal breast carcinomas (IDC) [48], depression and bipolar disorder [49], primary (PPMS) and secondary (SPMS) progressive multiple sclerosis [50], diabetes [51], Alzheimer’s disease [52], Huntington’s disease (HD) [53], and colorectal adenocarcinomas [54]. For example, the haplotypes rs10830963-rs4753426GC and rs10830963-rs4753426GT of *MTNR1B* were found to be associated with risk of PPMS and SPMS [51]. Homozygotes for the major allele, A, at rs10765576 of *MTNR1B* experienced a decreased risk of breast cancer compared to the GG or GA genotypes. Premenopausal women with the GG genotype were at increased risk for breast cancer compared with carriers of the major allele (TT or TG) for *MTNR1A* locus rs7665392, while postmenopausal women were at decreased risk [47].

Two point mutations (at exonic rs1202874 and intronic rs2072621) within, and the deletion of, the intracellular carboxyl terminus (*C*-tail) of the gene encoding *GPR50* have been shown to be associated with mental illnesses such as bipolar affective disorder (BPAD) and major depressive disorders [56]. The deletion of the more than 300 amino acid long *C*-tail of *GPR50* abolished the inhibitory effect of *GPR50* on *MTNR1A* function [56], presumably by preventing heterodimerization of the two proteins and more recent studies confirm that this deletion is associated with BPAD [57]. The variant *GPR50*Δ502–505at rs1202874 is in tight linkage disequilibrium with this deletion and was also found to be a sex-specific risk factor for susceptibility to bipolar disorder; other variants in the gene may be sex-specific risk factors in the development of schizophrenia [56]. An intronic variant at rs2072621 of this gene has been found to be associated with Seasonal Affective Disorder (SAD) in women [57]. As shown in Table 1, human *MTNR1B* exhibits more SNPs than *MTNR1A*, consistent with the greater pairwise distances between *MTNR1B* than *MTNR1A* sequences in Tables 3 and 4.

It seems to be of importance to not only link polymorphisms to diseases and disorders, but also to clarify the relationship between a risk factor and the changes in receptor function. This fundamentally important task is complicated by the fact that many of variants associated with a disease or disorder are also found in the general, nondiseased population and are sometimes nothing more than risk factors [58–60]. Substantial effects may be expected if receptors lose their high affinity to the ligand. However, many rodent strains, including numerous murine lab strains, are known to be melatonin-deficient. Defective melatonergic signaling may, thus, not be immediately apparent in an individual. Moreover, *MTNR1A* and *MTNR1B* can, to a certain degree, mutually substitute for each other, but not completely because of partially opposite effects and site-specific differences in signaling pathways [10,11]. Hence, even a knockout of one receptor subtype may be tolerable, what has occurred even in nature, as shown for Djungarian hamsters [61]. In humans, complete losses of melatonin binding and of expression at the cell surface were observed in the *MTNR1A* mutant I49N, and severe impairments in G166E and I212T [58]. No melatonin binding was described for the *MTNR1B* mutants A42P, L60R, P95L, and Y308S [59]. Despite poor surface expression and strongly reduced signaling towards G_i_-dependent adenylyl cyclase inhibition, G166E and I212T have partially retained their capability of activating the ERK1/2 pathway [58]. Thus, mutations can also cause changes in the coupling to alternate signaling pathways. Another example is the V124I mutant of *MTNR1B*, which is partially impaired with regard to the ERK1/2 but not the cAMP pathway [58]. Losses of G_i_-dependent signaling were described for 10 other *MTNR1B* mutants and a loss of ERK1/2 activation in R138C of the same gene [59]. Not only loss-of-function mutants may be unfavorable in terms of health, but this seems to be also possible for gain-of-function variants. Actually, the most frequently discussed example is that of the G allele of the *MTNR1B* SNP rs10830963, which is associated with a risk for diabetes type 2 and is now interpreted in terms of undesired overexpression in pancreatic β-cells [51].

Finally, it seems important to also investigate the consequences of changes in protein interaction domains of the receptors. As shown by site-directed mutagenesis, receptor affinity was neither altered by replacement of the palmitoylation site by alanine, nor by the truncation of the *C*-terminal domain, but the presence of both the lipid anchor and the *C*-terminal tail was required for G protein interaction [62]. Apart from this function, the *C*-terminal tail does not only contain phosphorylation sites, which are required for β-arrestin binding and formation of protein complexes involved in both signaling and internalization [27], but is also important for other protein-protein interactions. Another interaction partner at the *C*-tail of *MTNR1A*, but not of *MTNR1B*, is the PDZ domain protein MUPP1 (PDZ = PSD-95/*Drosophila* disc large/ZO-1 homology; MUPP1 = multi-PDZ domain protein 1) [63]. Binding of MUPP1 to *MTNR1A* did not alter localization or trafficking, but its disruption, by coexpression of PDZ fragments in HEK293 cells, abolished the cAMP response and gradually diminished ERK phosphorylation, which should have been stimulated by Gβγ, so that MUPP1 seems to be required for high-affinity binding of G_i_ to MT_1_. Moreover, the integrity of interaction domains required for *MTNR1A*/*MTNR1B* and *MTNR1A*/*GPR50* heterodimerizations as well as *MTNR1A* homodimerizations deserves future attention, since respective mutations will presumably alter the processes of regulation. This aspect even exceeds the mutual direct influences between the GPCR dimers, but seems to extend to interactions with members of the RGS (regulator of G-protein signaling) family. Several of its approximately 30 members have been reported to interfere with melatonergic signal transduction, such as RGS4 [64–66], RGS2 [66], and RGS20 [67]. A potentially important aspect has emerged from the RGS20 study, which led to the interpretation that an *MTNR1A* dimer binds to one monomer, the RGS, and to the other one, the G_i_ protein, which should have consequences to an effective RGS-mediated modulation. The complexity of interactions between melatonin receptors, *GPR50*, and RGS proteins may be higher than previously believed. Interestingly, silencing of RGS4 caused an upregulation of *GPR50* in a larger screen [68], findings that should, however, be confirmed by independent techniques.

## 4. Evolution of Melatonin Receptor Genes in Vertebrates

**Figure 2:**
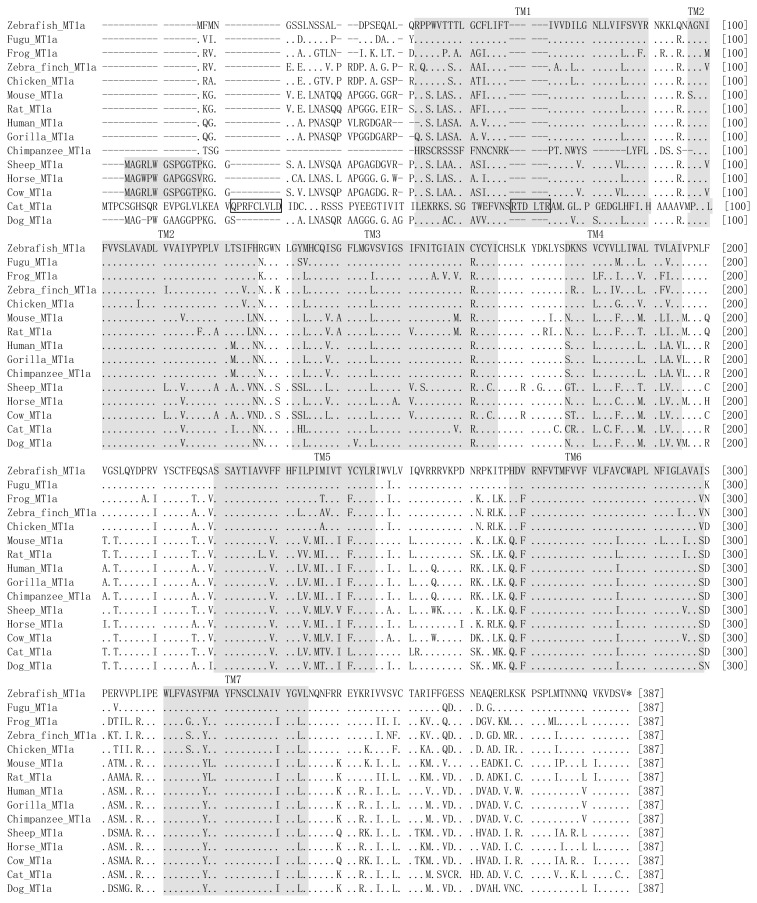
Sequence alignment of amino acids (AA) encoded by vertebrate *MTNR1A*; ‘.’ indicates the same AA, ‘-’ indicates an AA deletion. Two insertions of cat AA sequence are boxed.

The melatonin receptor and *GPR50* sequences (Table 2) were aligned by ClustalX [69] with manual adjustments. Neighbor-Joining trees (Figure 2) of 38 amino acid sequences (34 melatonin receptor sequences and 4 *GPR50* sequences) from 15 species were constructed in MEGA5 [70] using the Poisson correction method [71]. The reliability of branches of the estimated trees was evaluated by bootstrapping [72] with 1000 replications. Percentage bootstrap values are shown above branches in Figure 2.

We can see direct visualized differences of seven trans-membrane structures among human MT1A (classic 7 trans-membrane structure), cat MT1A (just have 6 trans-membrane structure) and human GPR50 (7 trans-membrane structure with a long tail) in Figure 1. Sequence alignment of amino acids encoded by *MTNR1A* in vertebrates shown in Figure 2 reveals two insertions in the N-tail and TM1 (trans-membrane 1) in cats. These two insertions cause cat *MTNR1A* to have only six trans-membrane domains (Figure 1, panel B). Physiological experiments are required to determine whether or not these insertions are associated with seasonal reproduction, nocturnality or any other phenotype.

The number of amino acid differences per site between sequences after eliminating gaps and missing data, calculated in MEGA5[67], are given below the diagonals in Tables 3 and 4 for *MTNR1A* and *MTNR1B*, respectively. *MTNR1B* exhibited generally higher values than *MTNR1A*, being consistent with the greater number of SNPs in human *MTNR1B* than human *MTNR1A*. Standard errors from 1000 bootstrap replicates, shown above the diagonal, ranged from 0.003 to 0.015, but most of them exceeded 0.010.

**Figure 3:**
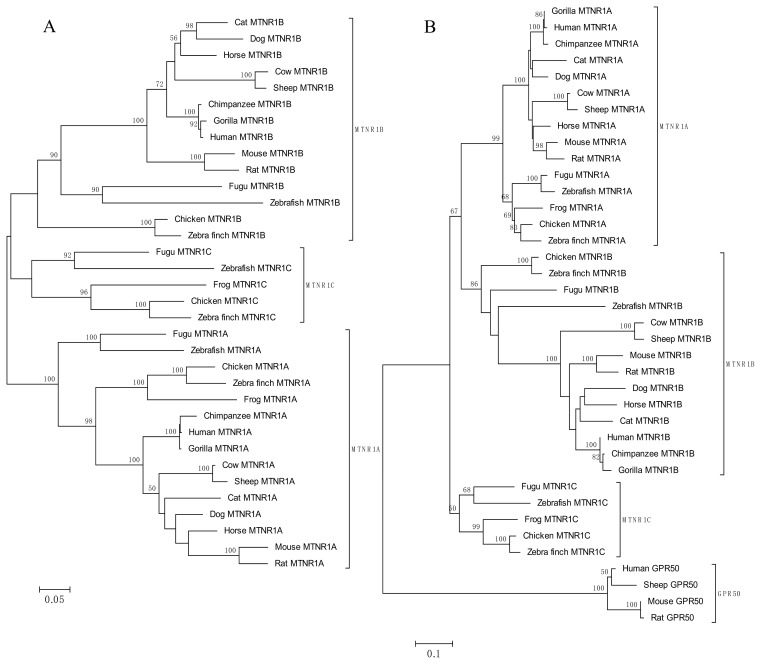
Neighbor-Joining (N-J) tree of melatonin receptors (panel **A**) constructed with protein Poisson distances; N-J tree of melatonin receptors and *GPR50* (panel **B**).

The Phylogenetic tree constructed from the amino acid sequences of 15 vertebrates, whose GenBank IDs were given in Table 2, is illustrated in Figure 3. The melatonin receptor of vertebrates is divided into three branches each representing a separate receptor subtype Figure 3A. The first split in the tree divide *MTNR1A* and *MTNR1C*, grouped with 69% bootstrap support, from *MTNR1B*, followed by the division between *MTNR1A*, with 100% support, and *MTNR1C*, with 63% support. When *GPR50* sequences are included (Figure 3B), all *GPR50* sequences, with 99% support, form an outgroup to all other melatonin sequences, followed by divergence of the *MTNR1C* sequences with 100% support from the *MTNR1A* and *MTNR1B* sequences. The next split in the tree divides all *MTNR1A* sequences, with 100% bootstrap support, from all *MTNR1B* sequences. Bootstrap support for all *MTNR1A* sequences, mammalian *GPR50* and *MTNR1B* sequences, and the *MTNR1C* sequences of lower vertebrates exceeded 99%. The *GPR50* gene was only detected in mammalian genomes (Table 2) while the *MTNR1C* gene was only detected in fish species, frogs and chicken genomes confirming previous results [5,73,74]. Sequence alignments encoded by the orthologous genes *MTNR1C* and *GPR50* reveal the addition of a long *C* terminal domain (Figure 1) in the *GPR50* receptor. As a consequence, the largest discrepancies between the sequence alignments of amino acids were observed for the *MTNR1C* and *GPR50* orthologs where sequence identity ranges from 45% to 79% [5]. Branch lengths, which reflect evolutionary time to common ancestral sequences [75], were clearly greater for the *GPR50* orthology group than for the other three groups suggesting that sequences from the *GPR50* group evolved earlier than *MTNR1A*, *MTNR1B* and *MTNR1C*, which are derived from a common ancestor and have rapidly differentiated from each other afterward.

## 5. Melatonin Receptors: A Perspective

Future research of melatonin receptors is promising under various aspects. With regard to melatonin’s unusually broad spectrum of actions [4], any deviations in receptor properties should cause a plethora of changes. This is already obvious from the polymorphisms detected to date and their associations with health problems. To better understand the consequences of the respective mutations, it will not be sufficient to identify losses or other alterations in agonist affinity, expression levels and surface localization. The multiple protein-protein interactions indicate that mutations can cause substantial deviations in regulation mechanisms. In terms of signaling pathways, differences between the receptor subtypes deserve further attention. On the other hand, the partial mutual substitution of *MTNR1A* and *MTNR1B* has to be considered, too. As mentioned, the natural knockout of the *MTNR1B* receptor gene in Djungarian hamsters does not alter seasonal reproductive and circadian responses [61]. Moreover, the targeted disruption of *MTNR1A* in mice has indicated that this receptor subtype is involved in melatonin’s suppressive action on SCN neurons, whereas *MTNR1B* is mainly required for phase shifting [76]. However, this conclusion is not necessarily valid for all species. In humans, *MTNR1B* is poorly expressed in the SCN [77]. Therefore, phase-shifting may be exerted via *MTNR1A*, which would require a sufficiently strong activation of protein kinase C by this subtype, and which is in other species, stimulated via *MTNR1B* [78]. A further intriguing question concerns the meaning of the greater conservation of *MTNR1A* than *MTNR1B* during vertebrate evolution, which may indicate a more profound role of the former relative to the latter in melatonin physiology. This would also be in line with the higher affinity of *MTNR1A* to melatonin. Nevertheless, the numerous associations of *MTNR1B* variants with health problems do require a thorough consideration of all properties of this subtype in humans.

## Figures and Tables

**Table 1:** Summary of polymorphisms of melatonin receptor genes in human.

	Location	Gene	Length	Amino acids length	Synonymous sites	Missense sites	Frame shift sites
Human	Chr: 4	*MTNR1A*	1053 bp	351	21	27	0
	Chr: 11	*MTNR1B*	1089 bp	363	18	50	0
	Chr: X	*GPR50*	1854 bp	618	9	21	3

**Table 2:** GenBank accession numbers of melatonin receptor and *GPR50* sequences.

	Species	Mel-1a GenBank ID	Mel-1b GenBank ID	Mel-1c GenBank ID	*GPR50* GenBank ID
Non-mammals	Zebrafish	NM_131393.1	NM_131395.1	NM_001161484.1	
	Fugu	AB492764.1	AB492765.1	AB492766.1	
	Frog	XP_002940910.1		U09561.1	
	Chicken	NM_205362.1	XM_417201.2	NM_205361.1	
	Zebra finch	NM_001048257.1	NM_001048258.1	XM_002193412.1	

Mammals	Horse	XP_001490221.1	XM_001917051.1		
	Gorilla gorilla	XM_004040725.1	XM_004051965.1		
	Cat	XM_003984615.1	XM_003992620.1		
	Human	NM_005958.3	NM_005959.3		NM_004224.3
	Chimpanzee	XM_526799.2	XM_522146.4		
	Rat	NM_053676.2	NM_001100641.1		NM_001191915.1
	Dog	XM_540019.3	XM_844629.2		
	Mouse	NM_008639.2	NM_145712.2		NM_010340.1
	Cow	XM_002698656.1	NM_001206907.1		
	Sheep	NP_001009725.1	NM_001130938.1		NM_001009726.1

**Table 3:** Estimates of *MTNR1A* evolutionary divergence (below diagonal) and standard errors (above diagonal) between sequences.

	Zebrafish	Cat	Chicken	Chimpanzee	Cow	Dog	Frog	Fugu	Gorilla	Horse	Human	Mouse	Rat	Sheep	Zebrafinch
Zebrafish		0.014	0.014	0.014	0.013	0.013	0.013	0.012	0.014	0.013	0.014	0.014	0.013	0.014	0.013
Cat	0.333		0.014	0.012	0.012	0.011	0.014	0.013	0.012	0.012	0.012	0.012	0.012	0.013	0.014
Chicken	0.276	0.286		0.014	0.012	0.012	0.012	0.013	0.013	0.013	0.013	0.013	0.013	0.013	0.009
Chimpanzee	0.302	0.182	0.289		0.013	0.012	0.014	0.013	0.009	0.012	0.008	0.012	0.012	0.013	0.014
Cow	0.297	0.188	0.260	0.210		0.011	0.013	0.013	0.011	0.011	0.011	0.012	0.011	0.005	0.012
Dog	0.281	0.153	0.219	0.196	0.146		0.013	0.012	0.010	0.009	0.010	0.010	0.010	0.011	0.012
Frog	0.287	0.310	0.211	0.315	0.271	0.258		0.013	0.013	0.013	0.013	0.013	0.013	0.014	0.012
Fugu	0.202	0.312	0.275	0.285	0.279	0.276	0.299		0.012	0.012	0.012	0.013	0.013	0.013	0.013
Gorilla	0.275	0.187	0.239	0.075	0.152	0.135	0.266	0.243		0.010	0.003	0.010	0.011	0.011	0.013
Horse	0.274	0.175	0.235	0.200	0.140	0.100	0.262	0.267	0.139		0.010	0.011	0.010	0.011	0.013
Human	0.276	0.187	0.239	0.074	0.152	0.138	0.268	0.244	0.007	0.137		0.011	0.011	0.011	0.013
Mouse	0.292	0.222	0.249	0.221	0.186	0.161	0.257	0.264	0.169	0.147	0.169		0.009	0.012	0.013
Rat	0.289	0.233	0.250	0.219	0.181	0.164	0.258	0.268	0.166	0.152	0.166	0.082		0.011	0.013
Sheep	0.292	0.202	0.258	0.216	0.029	0.150	0.276	0.279	0.159	0.145	0.159	0.192	0.187		0.013
Zebrafinch	0.276	0.282	0.102	0.296	0.256	0.235	0.210	0.269	0.251	0.244	0.249	0.263	0.265	0.258	

**Table 4:** Estimates of *MTNR1B* evolutionary divergence (below diagonal) and standard errors (above diagonal) between sequences.

	Zebrafish	Cat	Chicken	Chimpanzee	Cow	Dog	Fugu	Gorilla	Horse	Human	Mouse	Rat	Sheep	Zebrafinch
Zebrafish		0.014	0.014	0.014	0.014	0.016	0.014	0.014	0.014	0.014	0.014	0.014	0.014	0.015
Cat	0.367		0.015	0.011	0.011	0.012	0.014	0.011	0.011	0.011	0.012	0.012	0.012	0.015
Chicken	0.359	0.316		0.014	0.014	0.015	0.015	0.014	0.014	0.014	0.015	0.014	0.014	0.008
Chimpanzee	0.361	0.138	0.307		0.011	0.013	0.015	0.004	0.011	0.003	0.013	0.013	0.011	0.014
Cow	0.358	0.178	0.337	0.171		0.013	0.015	0.011	0.011	0.011	0.012	0.013	0.005	0.014
Dog	0.420	0.182	0.371	0.216	0.272		0.016	0.013	0.013	0.013	0.014	0.014	0.014	0.015
Fugu	0.329	0.338	0.347	0.334	0.339	0.389		0.014	0.015	0.014	0.014	0.015	0.014	0.015
Gorilla	0.362	0.139	0.306	0.019	0.170	0.221	0.333		0.011	0.003	0.013	0.013	0.012	0.013
Horse	0.364	0.133	0.303	0.131	0.173	0.210	0.325	0.133		0.011	0.012	0.012	0.011	0.014
Human	0.358	0.135	0.306	0.015	0.167	0.219	0.330	0.012	0.128		0.013	0.013	0.012	0.013
Mouse	0.381	0.192	0.338	0.190	0.240	0.288	0.357	0.191	0.201	0.191		0.009	0.013	0.015
Rat	0.380	0.200	0.340	0.202	0.250	0.284	0.358	0.203	0.206	0.204	0.095		0.013	0.014
Sheep	0.372	0.180	0.346	0.170	0.034	0.268	0.337	0.171	0.171	0.166	0.239	0.247		0.014
Zebrafinch	0.370	0.317	0.071	0.311	0.338	0.376	0.352	0.311	0.305	0.307	0.330	0.336	0.343	
